# Hepatoma-Derived Growth Factor: Its Possible Involvement in the Progression of Hepatocellular Carcinoma

**DOI:** 10.3390/ijms160614086

**Published:** 2015-06-19

**Authors:** Hirayuki Enomoto, Hideji Nakamura, Weidong Liu, Shuhei Nishiguchi

**Affiliations:** 1 Division of Hepatobiliary and Pancreatic Disease, Department of Internal Medicine, Hyogo College of Medicine, Mukogawa-cho 1-1, Nishinomiya, Hyogo 663-8501, Japan; E-Mail: nishiguc@hyo-med.ac.jp; 2 Department of Gastroenterology and Hepatology, Nissay Hospital, Itachibori 6-3-8, Nishi-ku, Osaka 550-0012, Japan; E-Mail: nakamura.hideji@nissay-hp.or.jp; 3 Department of Hepatology and Infectious Diseases, the Second Affiliated Hospital, Shantou University Medical College, No. 69, Dongxiabei, Jinping, Shantou 515041, China; E-Mail: weidongliu206@hotmail.com

**Keywords:** hepatoma-derived growth factor, hepatocellular carcinoma, angiogenesis, apoptosis

## Abstract

The development of hepatocellular carcinoma (HCC) is an important complication of viral infection induced by hepatitis virus C, and our major research theme is to identify a new growth factor related to the progression of HCC. HDGF (hepatoma-derived growth factor) is a novel growth factor that belongs to a new gene family. HDGF was initially purified from the conditioned medium of a hepatoma cell line. HDGF promotes cellular proliferation as a DNA binding nuclear factor and a secreted protein acting via a receptor-mediated pathway. HDGF is a unique multi-functional protein that can function as a growth factor, angiogenic factor and anti-apoptotic factor and it participates in the development and progression of various malignant diseases. The expression level of HDGF may be an independent prognostic factor for predicting the disease-free and overall survival in patients with various malignancies, including HCC. Furthermore, the overexpression of HDGF promotes the proliferation of HCC cells, while a reduction in the HDGF expression inhibits the proliferation of HCC cells. This article provides an overview of the characteristics of HDGF and describes the potential role of HDGF as a growth-promoting factor for HCC.

## 1. Introduction

The development of hepatocellular carcinoma (HCC) is a major complication of viral infection induced by hepatitis virus C [1,2]. HCC is a common malignant disease, and, despite recent progress in anticancer therapy, patients with advanced HCC continue to show a poor outcome [3,4]. Although several molecules have been determined to be potential targets of anticancer therapy [5], sorafenib, which was designed to inhibit vascular endothelial growth factor (VEGF) signaling, is the only available agent with a clinically demonstrated antitumor effect on HCC [6,7]. Therefore, identifying a new target molecule for HCC treatment is important.

We aimed to discover a new growth factor that may be involved in the progression of HCC. Since the hepatoma-derived cell line Huh-7 autonomously proliferates under serum-free conditions *in vitro*, we hypothesized the presence of an unknown growth factor in the conditioned medium and succeeded in purifying a new molecule, “hepatoma-derived growth factor (HDGF)” [8,9]. HDGF stimulates the proliferation of hepatoma cells *in vitro* [10,11], and the HDGF expression is significantly higher in human HCC tissues than in adjacent non-cancerous liver tissues [12]. A high HDGF expression is related to several unfavorable cancer characteristics, including rapid growth, significant invasiveness and metastasis, and it is also associated with poor prognoses of various malignant diseases [13,14,15,16,17,18,19].

We initially identified HDGF as a growth-stimulating factor; however, HDGF has also been reported to be an angiogenic factor and probable anti-apoptotic factor. Therefore, this novel molecule may participate in the development and progression of many types of cancer through multiple mechanisms. This article reviews the characteristics of HDGF and describes the potential role of HDGF as a unique growth-promoting factor for HCC.

## 2. HDGF as a Novel Unique Growth Factor

HDGF is a 26-kDa heparin-binding acidic glycoprotein consisting of 240 amino acids that was originally reported as a secreted protein purified from the conditioned medium of Huh-7 hepatoma cells [8,9]. Several novel proteins, the N-terminal regions of which are highly homologous to that of HDGF, were subsequently identified, named HDGF-related proteins (HRPs) [20,21,22]. HDGF and HRPs are considered to form a new gene family, and the highly homologous N-terminal region containing approximately 100 amino acids is represented as the HATH (homologous to the amino terminus of HDGF) domain. Additionally, a survival factor for the lens epithelium, LEDGF (lens epithelium-derived growth factor) [23], is also suggested to be a member of the HDGF family based on the presence of a HATH region in its N-terminus.

Although HDGF proteins are detected in the conditioned media of various types of cells, the sequence of the HDGF protein does not include the N-terminal hydrophobic sequence characteristic of signal peptides. Therefore, HDGF is assumed to be secreted via a pathway that differs from the classical Golgi secretion system [9,24]. A recent study showed that the 10 amino acids at the N-terminus of HDGF are essential for its secretion. It has also been reported that the phosphorylation of serine 165 in the C-terminal region of HDGF is important for its secretion [25].

Irrespective of the unclarified system(s) of secretion, the exogenous administration of HDGF stimulates the proliferation of various cell types, including benign and malignant cells [26,27,28,29,30,31]. In addition, the exogenous administration of HDGF enhances the phosphorylation of mitogen-activated protein kinase (MAPK) in gastric epithelial cells and endothelial cells [32,33]. Furthermore, exogenously supplied HDGF proteins activate phosphatidylinositol-3 kinase (PI3K)/AKT signaling in NIH3T3 cells [34]. These findings strongly suggest the presence of receptor-mediated signal transduction pathway(s) for HDGF. Recently, part of the HATH region (amino acids 81–100) has been reported to be a possible receptor-binding site [35]. Therefore, the growth-promoting effects of HDGF should at least partially depend on the receptor-mediated signal transduction pathways, such as the intracellular activation of MAPK and/or PI3K/AKT.

**Figure 1:**
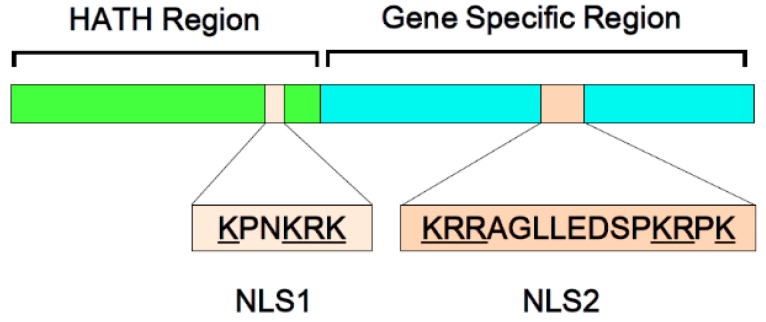
Structure of hepatoma-derived growth factor (HDGF). HDGF protein contains 240 amino acids. The N-terminal region of HDGF protein is highly homologous to that of HDGF-related proteins, and the well-conserved N-terminal amino acid sequence (approximately 100 amino acids) is represented as the HATH (homologous to the amino terminus of HDGF) domain. HDGF has two putative nuclear localization signals (NLSs). The first NLS is a basic amino acid-rich region (^75^ **K**PN**KRK** ^80^; basic residues underlined) in the HATH domain (NLS1), and HDGF protein also contains a basic motif (^155^ **KRR**AGLLEDSP**KR**P**K** ^170^; basic residues underlined) in the gene-specific region (NLS2).

In addition to the role of HDGF in receptor-mediated signal transduction, HDGF has two putative nuclear localization signals (NLSs) and it can be transported into the nucleus, suggesting that it may function as a nuclear factor [10,26]. The first NLS is located in the HATH region, while the second NLS resides in a gene-specific region (Figure 1). We previously found that nuclear translocation is important for the mitogenic activity of HDGF-overexpressing cells and that the second NLS plays a pivotal role in the growth-stimulating effects of HDGF [10].

Although it is unclear how HDGF stimulates cellular growth after nuclear translocation, previous studies have suggested major roles for the HATH region. The HATH regions of the HDGF family members contain a PWWP motif [36,37] that was initially reported in a candidate gene for Wolf-Hirschhorn syndrome, WHSC1. HDGF binds to a conserved DNA sequence in the promoter region of its target genes and subsequently inhibits their transcription, and the presumed DNA binding site is suggested to be present in the PWWP domain [38]. Therefore, the PWWP motif in the HATH region of HDGF has the potential to function as a DNA binding domain.

**Figure 2:**
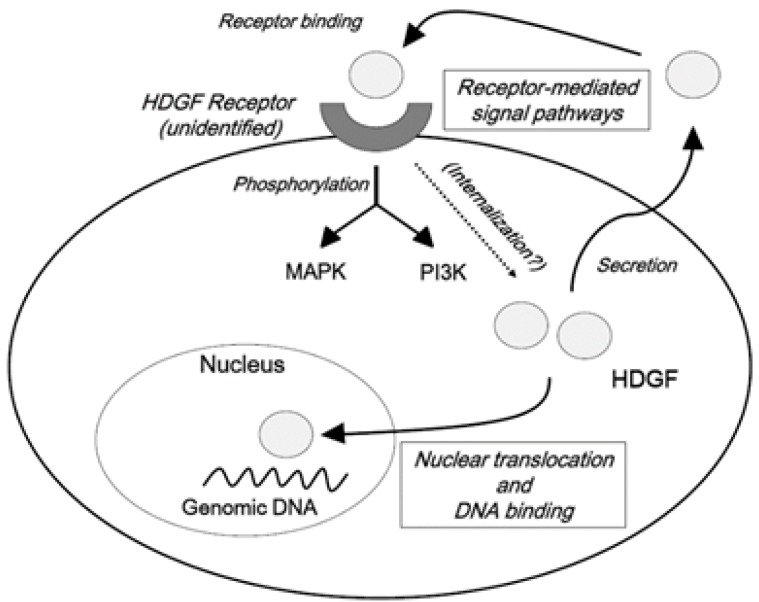
Possible signal pathways of hepatoma-derived growth factor (HDGF). HDGF is a unique growth factor with dual mechanisms for promoting cellular proliferation: a receptor-mediated pathway and a direct action mediated by DNA binding following nuclear translocation. HDGF is considered to be secreted via a pathway that differs from the classical Golgi secretion system. Extracellular HDGF binds to an unidentified receptor and activates MAPK and/or PI3K. HDGF protein also acts as a nuclear protein that regulates the expression of target genes through DNA binding.

Taken together, HDGF is a unique growth factor with dual mechanisms for promoting cellular proliferation: a receptor-mediated pathway and a direct action mediated by DNA binding following nuclear translocation (Figure 2).

## 3. HDGF as an Angiogenic Factor

Although HDGF was isolated from a hepatoma cell line, previous studies have shown that it plays important roles in the development and tissue repair of numerous normal organs, including the liver, kidneys, lungs and gut [27,28,29,30,31]. In addition, Everett *et al.* [39] reported the functional involvement of HDGF in the development of and tissue repair in the cardiovascular system. We have also shown that HDGF stimulates the proliferation of endothelial cells and that the administration of recombinant HDGF significantly increases tubular formation in experimental vascular formation systems *in vitro* [33,40]. Interestingly, we further demonstrated that the introduction of the HDGF gene in NIH3T3 fibroblasts induces the expression of the most important angiogenic factor VEGF and that the transplantation of HDGF-overexpressing NIH3T3 cells in nude mice results in the development of large and highly vascularized tumors [40]. VEGF is highly expressed in the tumors developing from these HDGF-overexpressing NIH3T3 cells, and the growth of HDGF-overexpressing tumors is partially inhibited by an anti-VEGF neutralizing antibody [40]. In agreement with the findings of our study, HDGF has also been reported to induce VEGF in a gastric cancer cell line [41]. These findings suggest that HDGF may function as an angiogenic factor through two different mechanisms, including its direct effect on endothelial cell proliferation and the induction of VEGF expression.

## 4. HDGF as a Possible Anti-Apoptotic Factor

The ability to escape apoptotic signals is one of the fundamental mechanisms underlying the survival and uncontrolled growth of malignant cells, and several growth factors are suggested to participate in tumor progression via their anti-apoptotic effects [42]. However, the role of HDGF in the apoptotic pathway remains controversial.

HDGF expression is considered to be related to the increased sensitivity of esophageal cancer cells to radiation therapy [43]. Additionally, the dephosphorylation of HDGF may be involved in caspase-dependent apoptosis under the TNF/cycloheximide-induced apoptosis of endothelial cells [44]. The reduction of HDGF inhibits TNF/cycloheximide-induced apoptosis in HeLa cells [45]. In contrast, several studies have shown an association between HDGF and apoptotic resistance, rather than the induction of apoptosis [46,47,48,49,50,51]. For instance, HDGF has been reported to be a survival factor for neurons of the central nervous system, motor neurons and olfactory epithelium [46,47]. The reduction of HDGF induces the expression and dephosphorylation of the pro-apoptotic protein Bad and suppresses the ERK-AKT signaling of MAPK, resulting in the activation of the apoptotic pathway [48]. In colorectal cancer cells, the overexpression of HDGF inhibits drug-induced apoptosis, and HDGF knockdown induces apoptosis through the mitochondrial pathway, suggesting that HDGF is involved in the process of cancer cell resistance to chemotherapy [49,50]. Regarding hepatoma cells, blocking HDGF activates both the Fas-mediated extrinsic and Bad-mediated intrinsic apoptotic pathways [48,51], and HDGF is therefore considered to function as a survival factor by exerting multiple anti-apoptotic effects. However, further studies are required before any definitive conclusions can be made regarding the functional role of HDGF in apoptosis, although most recent reports support the anti-apoptotic function of HDGF in various types of cancer cells.

## 5. HDGF as a Growth-Promoting Factor for HCC

HDGF is expressed in several hepatoma cell lines, including Huh-7, HepG2, PLC/PLF/5, SK-Hep1 and Mahlavu [52,53]. The endogenous overexpression of HDGF significantly stimulates the proliferation of hepatoma cells [13]. In a xenograft model using nude mice, HDGF-overexpressing HepG2 hepatoma cells develop larger tumors when compared to the tumors of the control counterparts [52]. In clinical studies, the expression of HDGF in human HCC tissue has been found to be higher than that seen in adjacent non-cancerous tissues [12]. Additionally, HCC patients with a higher HDGF expression have been reported to show earlier recurrence and a poorer overall survival rate compared to patients with lower HDGF expression levels [15,54,55]. A multivariate analysis revealed that high HDGF expression and serum AFP (a well-established tumor marker) levels were independent prognostic factors for the disease-free and overall survival [15]. These findings suggest the significant role of HDGF in the progression of human HCC.

As described above, the HDGF expression has been suggested to contribute to the progression of various malignant diseases. Treatment with HDGF antibodies significantly suppresses the proliferation of lung cancer cells, making HDGF a novel therapeutic target for lung cancer [56]. Meng *et al.* investigated the effects of HDGF-silencing through shRNA and reported that HDGF may be a therapeutic target for non-small cell lung cancer [57]. Furthermore, several recent experimental studies showed HDGF to be associated with the malignant phenotype of cancer cells [58,59,60]. We previously showed that the reduction of the HDGF expression significantly suppresses the growth of hepatoma cells *in vitro* [11,53]. We recently found that the reduced expression of HDGF inhibits the proliferation of HCC cells *in vivo* [61]. Since a high HDGF expression is significantly associated with the unfavorable prognosis of various malignant diseases in clinical studies, it is possible that the inhibition of HDGF will provide a new approach for the treatment of malignant diseases, including HCC.

## 6. Future Perspectives

Although HDGF is considered to be a unique growth factor and a potential target molecule for anticancer therapy, the mechanisms by which the HDGF expression is inhibited remain unclear. As a result, more studies must be carried out to obtain new knowledge that is required to develop reliable therapies. Meanwhile, several studies have suggested various mechanisms for regulating the HDGF gene expression. We previously showed that vitamin K2 inhibits the promoter activity of HDGF in hepatoma cells [53]. HDGF may be negatively regulated by the anticancer protein p53 [62]. Interestingly, Shih *et al.* showed that HDGF is a target gene of microRNA (miR)-214 and that the downregulation of miR-214 contributes to the hypervascularity of HCC via activation of the HDGF paracrine pathway for tumor angiogenesis [63]. In addition, other microRNAs have been reported to affect the HDGF expression. For example, the HDGF expression is upregulated by miR-195 or miR-497 in lung cancer cells [64,65], and miR-141 induces the HDGF expression in gastric cancer cells [66]. Interestingly, using affinity chromatography and proteomic techniques, nucleolin has recently been reported to be a HDGF-interacting membrane protein in hepatoma cells [67].

Nevertheless, the mechanisms underlying the regulation of the HDGF expression and signal transduction must be better understood in order to establish viable anti-HCC strategies.

**Figure 3:**
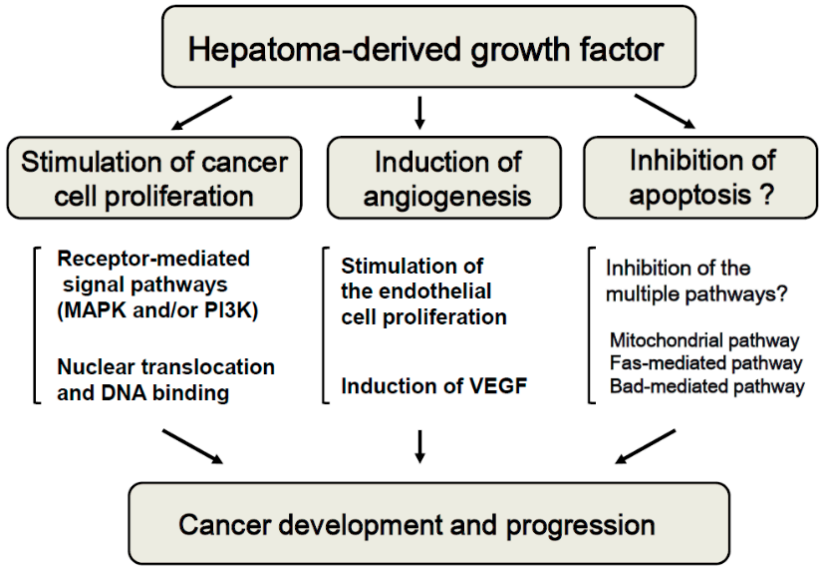
Roles of hepatoma-derived growth factor (HDGF) in the development and progression of malignant diseases. HDGF is a unique multi-functional protein that can function as a growth factor, angiogenic factor and anti-apoptotic factor and it participates in the development and progression of malignant diseases, including HCC.

## 7. Conclusions

HDGF is a unique molecule with multiple cellular roles. For example, it functions as a growth-stimulating factor, angiogenic factor and possible anti-apoptotic factor (Figure 3). Recent studies have demonstrated the overexpression of HDGF to correlate with poor clinical outcomes in numerous types of malignant diseases. Understanding the regulation of HDGF will thus provide a path for developing novel therapies against HCC.

## Abbreviations

HCC: hepatocellular carcinoma
VEGF: vascular endothelial growth factor
HDGF: hepatoma-derived growth factor
HRP: HDGF-related protein
HATH: homologous to the amino terminus of HDGF
MAPK: mitogen-activated protein kinase
PI3K: Phosphatidylinositol-3 kinase
NLS: nuclear localization signal
NSCLC: non-small cell lung cancer
miR: microRNA
